# Association of a novel point mutation in MSH2 gene with familial multiple primary cancers

**DOI:** 10.1186/s13045-017-0523-y

**Published:** 2017-10-03

**Authors:** Hai Hu, Hong Li, Feng Jiao, Ting Han, Meng Zhuo, Jiujie Cui, Yixue Li, Liwei Wang

**Affiliations:** 1 Department of Oncology, State Key Laboratory for Oncogenes and Related Genes, Renji Hospital, School of Medicine, Shanghai Jiaotong University, Shanghai Cancer Institute, Shanghai, 201620 China; 2 Key Lab of computational Biology, CAS-MPG Partner Institute for Computational Biology, Shanghai Institutes for Biological Sciences, Chinese Academy of Sciences, Shanghai, 200031 China; 3 Shanghai Center for Bio information Technology, 1278 Keyuan Road, Shanghai, 201203 China; 4 Department of Medical Oncology and Pancreatic Cancer Center, Shanghai General Hospital, School of Medicine, Shanghai Jiao Tong University, Shanghai, 201620 China

**Keywords:** Lynch syndrome, Cancer landscape, MSH2, Carcinogenesis, Multiple primary cancers

## Abstract

**Background:**

Multiple primary cancers (MPC) have been identified as two or more cancers without any subordinate relationship that occur either simultaneously or metachronously in the same or different organs of an individual. Lynch syndrome is an autosomal dominant genetic disorder that increases the risk of many types of cancers. Lynch syndrome patients who suffer more than two cancers can also be considered as MPC; patients of this kind provide unique resources to learn how genetic mutation causes MPC in different tissues.

**Methods:**

We performed a whole genome sequencing on blood cells and two tumor samples of a Lynch syndrome patient who was diagnosed with five primary cancers. The mutational landscape of the tumors, including somatic point mutations and copy number alternations, was characterized. We also compared Lynch syndrome with sporadic cancers and proposed a model to illustrate the mutational process by which Lynch syndrome progresses to MPC.

**Results:**

We revealed a novel pathologic mutation on the MSH2 gene (G504 splicing) that associates with Lynch syndrome. Systematical comparison of the mutation landscape revealed that multiple cancers in the proband were evolutionarily independent. Integrative analysis showed that truncating mutations of DNA mismatch repair (MMR) genes were significantly enriched in the patient. A mutation progress model that included germline mutations of MMR genes, double hits of MMR system, mutations in tissue-specific driver genes, and rapid accumulation of additional passenger mutations was proposed to illustrate how MPC occurs in Lynch syndrome patients.

**Conclusion:**

Our findings demonstrate that both germline and somatic alterations are driving forces of carcinogenesis, which may resolve the carcinogenic theory of Lynch syndrome.

**Electronic supplementary material:**

The online version of this article (10.1186/s13045-017-0523-y) contains supplementary material, which is available to authorized users.

## Background

Multiple primary cancers (MPC) have been defined as two or more cancers without any subordinate relationship that occurs either simultaneously or metachronously in the same or different organs of an individual [1]. Since Billroth proposed the concept of MPC in 1889 [2], researchers had been attracted by the disease and emerging patients have been identified [3–6] owing to advanced diagnostic technologies [7–9], sustained environmental degradation [10], and longer life expectancies of cancer survivors [11, 12]. To date, studies on MPC were mainly descriptive with little investigating of the mechanism whereby MPC occurs [13, 14]. Hence, it is of great urgency to learn the machinery whereby MPC occurs so as to provide prevention strategies in the future.

In clinical settings, many MPC patients had been proven to have a strong family history of cancer, while others were sporadic. Lynch syndrome is a dominant genetic disorder characterized by an increased risk of cancers of the digestive tract, gynecologic tract, and other organs [15]. Germline mutations of DNA mismatch repair (MMR) genes including MLH1 (42%), MSH2 (33%), MSH6 (18%), and PMS2 (7%) and several less-frequent genes (PMS1, MSH3, and EPCAM) are the major causes of Lynch syndrome [16]. Mutated MMR genes are not able to repair DNA replication errors. As cells with that specific defect continue to divide, the mistakes accumulated and usually led to cancers. Therefore, it is common that Lynch syndrome patients usually suffer more than two cancers. Strategically, Lynch syndrome patients who suffer more than two cancers provide a unique resource to study the pathogenesis of MPC.

Previously, increasing studies had focused on MPC, but most of which were descriptive with none presented with pronounced and compelling illustrations on how MPC occurs [17–21]. As to Lynch syndrome, most mechanistic studies were focused on MMR genes. To the best of our knowledge, no studies had systematically investigated the mutational landscape when Lynch syndrome progressed to MPC. In the past decades, the “omics” studies had achieved many discoveries in various human malignancies, which opened a new window for understanding cancer initiation and progression. Lynch syndrome, together with genomic study strategies, provides a unique avenue to investigate the carcinogenic mechanism of MPC.

In the present study, we performed a comparative genome analysis on peripheral blood cells and two primary tumors of a patient with Lynch syndrome. We discovered a novel MSH2 mutation (G504 splicing) associating with Lynch syndrome, which segregated with disease phenotypes in a four-generational pedigree and resulted in the inactivation of MSH2 protein. Systematical comparison of somatic point mutations and copy number alterations revealed that these two cancers were evolutionarily independent. We further demonstrated that Lynch syndrome-related cancers harbored mutations in the driver genes of sporadic cancers, and that these genes might play significant roles in the carcinogenesis of Lynch syndrome. Furthermore, a model was proposed to illustrate how Lynch syndrome progressed to MPC.

## Methods

### Patients and clinical samples

The study included eight subjects in total, and they are from a single family. Of all the subjects, four are cancer patients, while the rest were healthy. All the patients were still alive with excellent physical conditions and received surgical resection at Shanghai General Hospital Affiliated Shanghai Jiaotong University, School of Medicine. The formalin-fixed, paraffin-embedded (FFPE) cancer tissues were collected from the Department of Pathology. The medical records, together with 2 mL anticoagulant blood of all the subjects, were collected in our clinics. Detailed information of the patients are summarized in Addtional file 1: Table S1. Written informed consents and approval by the Ethics Committee of Shanghai General Hospital were obtained for the use of these clinical materials for research purposes.

### Whole genome sequencing

The whole genome sequencing was performed on three samples from the proband: Blood, FFPE samples of renal pelvic carcinoma (RPC), and small intestine cancer (SIC). Genomic DNA was extracted using Qiagen DNeasy Kits (Cat No. /ID56404 for FFPE tissues and Cat No. /ID51104 for blood samples) according to the manufacturer’s instructions. A sequencing library was constructed from 500 ng genomic DNA using a TruSeq Nano DNA Sample Prep kit. A DNA library was sequenced on an Illumina X Ten platform using 2 × 150 base pair (bp) paired-end reads.

### Analysis of sequence data

The raw sequencing reads were filtered by in-house programs, removing low-quality reads with greater than 10% uncalled bases, trimming N bases at the end of reads, and removing chimera that had greater than 15 bases matched to the primer sequences. Reads that passed quality control were mapped to human genome (hg19) by BWA v0.7.12 [22]. Duplicated sequences were masked by Picard tools v1.136. Genome Analysis Toolkit (GATK v 3.4-46) [23] was used to call single nucleotide variants (SNVs) and short insertions/deletions (indels). SNVs and indels were separately filtered by the recommended parameters in GATK best practice. Functional effects of variants were annotated by ANNOVAR software [24]. Somatic mutations were comprehensively identified by VarScan (v2.3.9) [25], MutTect (v1.1.7) [26], SomaticSniper (v1.0.4) [27], and Strelka (v 1.0.14) [28] with default parameters. To avoid false positive data, we only retained mutations that were identified by more than one software program and removed potential germline variants whose frequencies were greater than 1% in dbSNV 138 [29]. To identify somatic copy number alterations (SCNAs), we selected high-quality germline SNVs meeting the following criteria: (1) identified in all samples of the same patient, with coverage greater than 20; (2) dbSNP entry and heterozygous; and (3) minor allele frequency (MAF) in the normal sample was at least 0.25. Then, we plotted the MAF values in windows of 1000 SNVs with 500 SNVs overlap. We compared the MAF curve between tumor and normal samples to identify arm-level SCNAs [30].

### Validation of MSH2 mutations

To evaluate MSH2 germline mutations in other family members, the blood cells of eight members were collected and the DNA were extracted. Polymerase chain reaction (PCR) and Sanger sequencing were utilized to check the genetic profile of MSH2 gene. The standard protocol for PCR and Sanger sequencing has been described elsewhere [31–33]. The genomic region surrounding the MSH2 mutation site was subjected to PCR amplification and cloned into the pEASY vector (Transgen), which was used for Sanger sequencing (JieRui, Shanghai, China). A total of 200–400 ng PCR products (TakaRa) were subjected to a reannealing process to enable heteroduplex formation: 95 °C for 10 min, 95 to 85 °C ramping at 2 °C/s, 85 to 25 °C at 0.3 °C/s, and holding at 25 °C for 1 min. After that, products were treated with SURVEYOR nuclease and SURVEYOR enhancer S (Transgenomic), following the manufacturer’s instructions, and analyzed on 10% polyacrylamide gels. Gels were stained with 0.5 μg/mL ethidium bromide in 1 × Tris/Borate/EDTA for 20 min, washed in water for 20 min and imaged with a gel-imaging system (Tanon). Quantification was based on band intensity. The primer used in the analysis was F: GATGGGTTTACCCAGAAAGCAG, R: TCATGTTAGAGCATTTAGGGA.

### IHC

The standard protocol for immunohistochemistry (IHC) has been described previously [34]. Briefly, tissue sections were dewaxed and dehydrated in a xylene and alcohol bath solution. Endogenous peroxidase activity was blocked by incubation in 0.3% hydrogen peroxide. Antigen retrieval was achieved by incubating the slides in 0.01 M citrate buffer (pH 6.0) at 98 °C for 5 min using a microwave oven. The slides were cooled to and blocked in normal goat serum at room temperature for 1 h, followed by incubation with the primary antibody MSH2 (CST) at 4 °C overnight. The sections were incubated with a horseradish peroxidase-labeled secondary antibody and visualized using 3, 3′-diaminobenzidine.

### Evaluation of IHC

Two independent pathologists blind to the study performed IHC evaluation. Five visual fields from different areas of each specimen were chosen at random for evaluation. The expression was scored according to the staining intensity and the percentage of positive cells [35]. The percentage of positive cells was scored as follows: 0% (0), 1%–10% (1), 11%–50% (2), and 51%–100% (3). Staining intensity was scored as follows: no staining (0), weak (1), moderate (2), and strong staining (3). The final scores were calculated by the staining intensity × the percentage of positive cells. For statistical analyses, scores less than six were regarded as negative, while the rest were positive.

### H&E staining

To confirm the clinical diagnosis of the malignancies, hematoxylin and eosin (H&E) staining was performed on the samples. Briefly, tumor samples were fixed with paraformaldehyde and embedded in paraffin. The paraffin blocks were sliced into 5-μm-thick sections and mounted onto glass microscope slides. Subsequently, the slides were deparaffinized using xylene and a graded series of alcohol prior to being stained with H&E. Five randomly selected microscopic fields from each slide were examined under a microscope by two pathologists blind to the study.

### Integrative data analysis

To investigate the difference of two cancers (RPC and SIC) in the proband, we compared the SNVs and SCNAs. We focused on cancer-related gene lists, including 127 significantly mutated genes (SMGs) in The Cancer Genome Atlas (TCGA) Pan-Cancer analysis [35], 572 genes in the Cancer Gene Census [36], and genes in 13 cancer signaling pathways [37]. In addition, Lynch syndrome was compared to cancers in TCGA. Somatic mutations of TCGA Pan-Cancer analysis were downloaded from Synapse (https://www.synapse.org/#! Synapse: syn1729383). Clinical data of TCGA patients were retrieved from BROAD GDAC Firehose (http://gdac.broadinstitute.org/). TCGA cancer samples were classified into three groups based on the results of the microsatellite instability (MSI) test: MSI high (MSI-H), MSI low (MSI-L), and microsatellite stable (MSS). MSI was often observed in four cancer types: colon adenocarcinoma, rectum adenocarcinoma, uterine corpus endometrial carcinoma, and stomach adenocarcinoma. We also selected another four cancers without MSI samples: ovarian serous cystadenocarcinoma, breast invasive carcinoma, glioblastoma multiform, and kidney renal clear-cell carcinoma. Previous studies have reported many MSH2 germline mutations associated with Lynch syndrome. The mutation sites were collected from the ClinVar database [38] and were compared to MSH2 somatic mutations in TCGA cancers. All statistical analyses were performed with R version 3.2.2.

## Results

### A Chinese family with Lynch syndrome

**Fig. 1:**
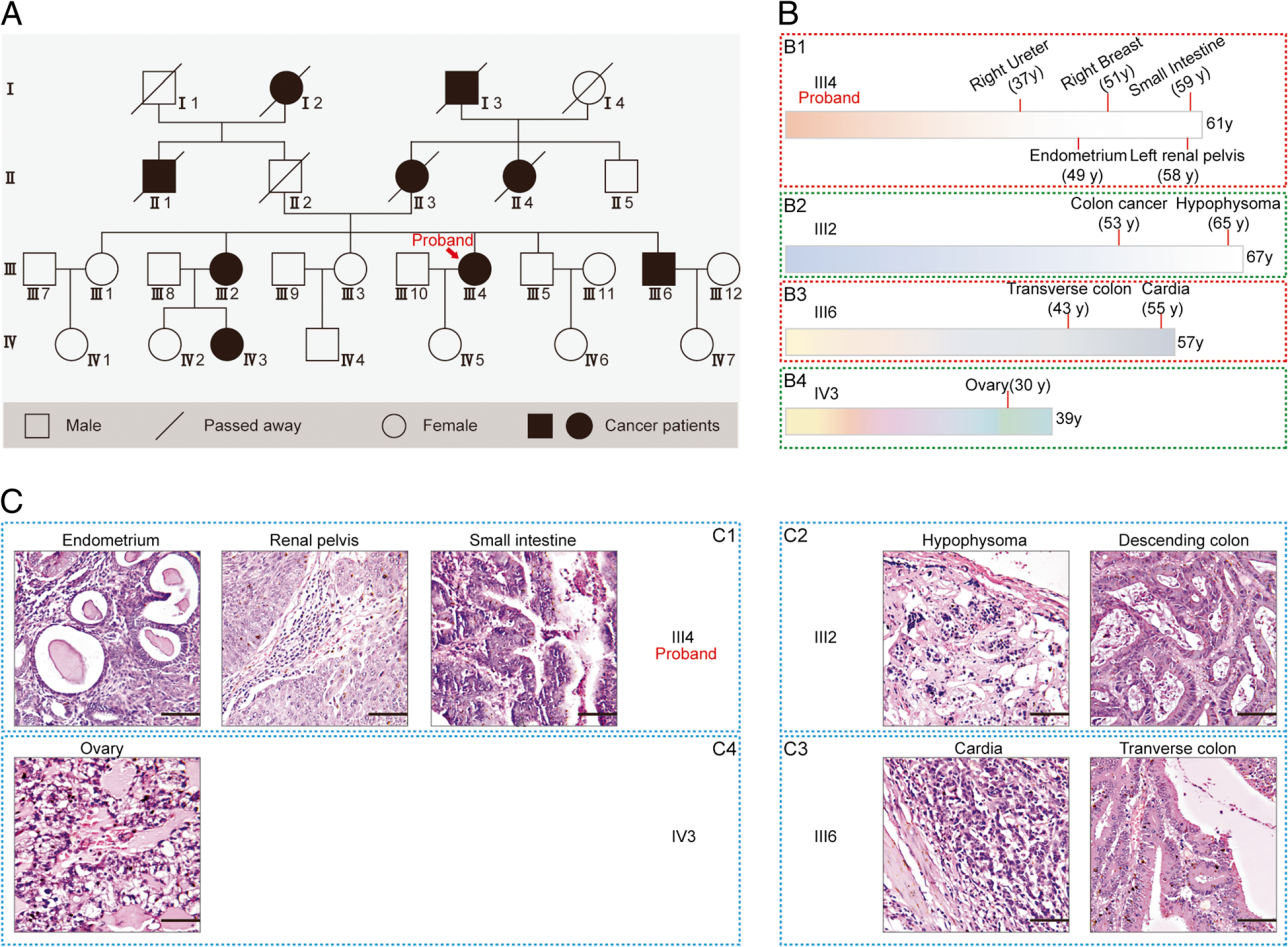
A Chinese family with Lynch syndrome. **a** The pedigree of the proband. The arrow indicates the proband (III4). Circles and squares denote females and males, respectively. Filled symbols indicate patients with MPC or a single cancer. Empty symbols define unaffected individuals. **b** Brief summary of the medical history of each patient. **c** Representative images of hematoxylin and eosin staining of the cancers. Scale bar 200 μm

The proband (III4) in the study was a quintuple primary cancer patient, namely right ureteral transitional cell papilloma, left breast infiltrative ductal carcinoma, endometriosis type adenocarcinoma (Fig. 1 B1 and C1), left renal pelvic infiltrating urothelial carcinoma (Fig. 1 B1 and C1), and small intestine ulcerative infiltrative adenocarcinoma (Fig. 1 B1 and C1).

A family survey on the proband showed that nine members in four consecutive generations suffered malignancies (Additional file 1: Table S1, Fig. 1a). Patients from the third (III) and fourth (IV) generations were still alive with excellent physical conditions, and the malignancies were confirmed by postoperative pathologies. Patient III2 suffered ascending colon papillary adenocarcinoma and hypophysoma (Fig. 1 B2 and C2); III6 suffered transverse colon tubular adenocarcinoma and poorly differentiated cardia carcinoma (Fig. 1 B3 and C3), and IV3 had ovarian cancer (Fig. 1 B4 and C4). Patients from the first (I) and second (II) generations all died. Specifically, I2 died of cervical cancer in 1961; I3 died of esophageal cancer in 1957; II1 died of nasopharyngeal carcinoma in 1965; II3 died of malignant glioma in 1968; and II4 died of esophageal cancer in 2002.

Comparison between the pedigree and Amsterdam criteria II, which has been widely applied to aid the diagnosis of Lynch syndrome [39], suggested that this family could be diagnosed as having Lynch syndrome if they were excluded from familial adenomatous polyposis (FAP). In fact, patients who were still alive were all proved to be FAP-negative by colonoscopy during the regular follow-up. Therefore, the patients could be diagnosed as having Lynch syndrome, and patients with more than two tumors could be treated as unique MPC. Then, we carried out whole genome sequencing to reveal the mutational landscape of this unique disease.

### Causal variant of Lynch syndrome

**Fig. 2:**
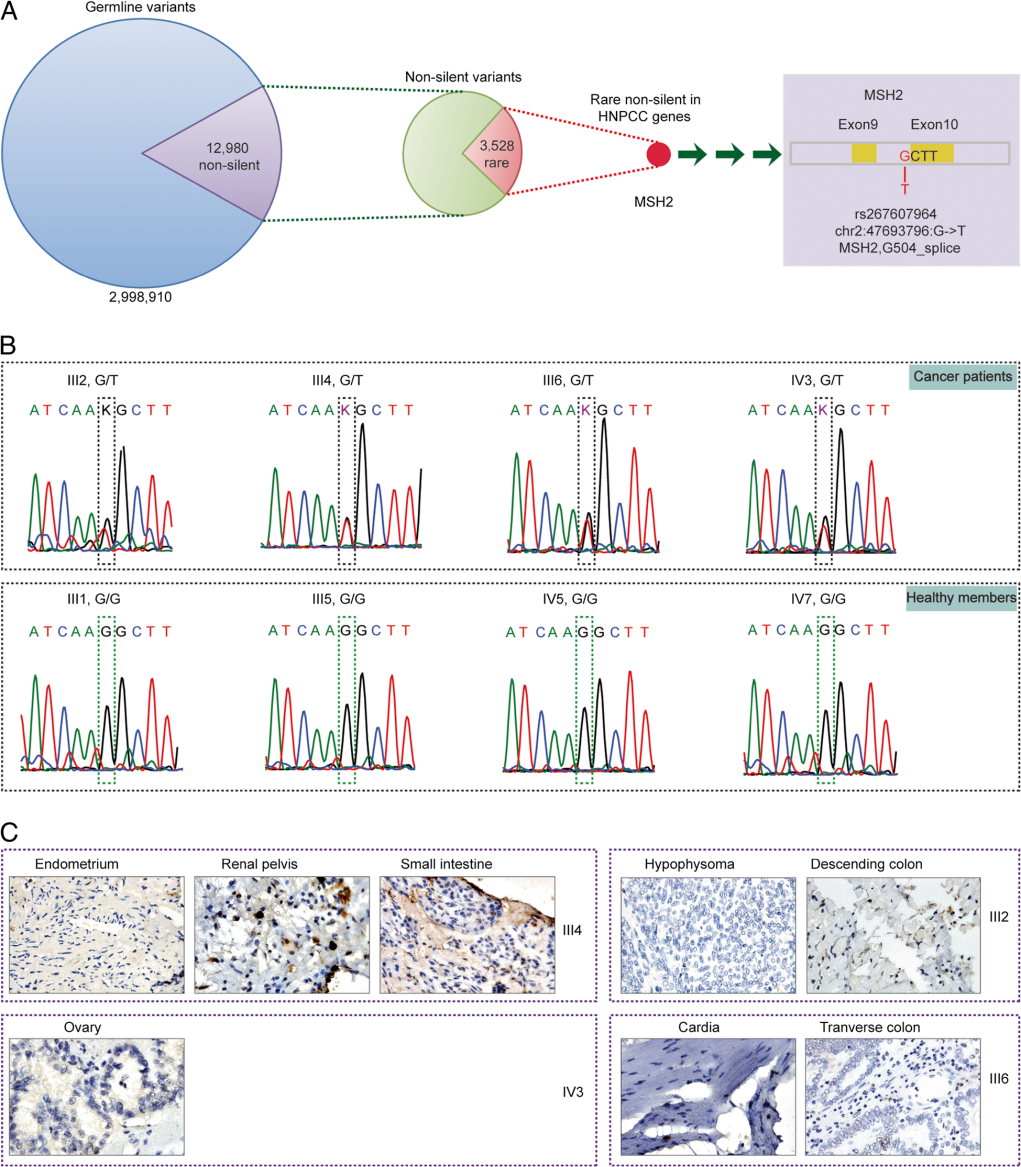
Germline mutation and inactivation of MSH2 in a family with Lynch syndrome. **a** Identification of a causal germline mutation in MSH2 from whole genome sequencing. **b** Validation of MSH2 mutation in affected and unaffected members. All affected individuals have an rs267607964 variant, while unaffected individuals do not have such mutation. **c** The detection of MSH2 expression in protein levels in cancer tissues

We analyzed the blood cells, renal pelvic carcinoma (RPC), and small intestine cancer (SIC) samples of the proband (III4) using whole genome sequencing. On average, we obtained 37 × coverage, and we identified approximately 3.4 million single nucleotide variants (SNVs) and 0.7 million indels in each sample (Additional file 1: Tables S2, S3). After we analyzed the samples, we obtained 2,998,910 (361,948) overlapping germline SNVs (indels). The major variants were located in intergenic or intron regions, while 12,980 (903) non-silent SNVs (indels) might cause protein functional changes. Since the incidence of hereditary non-polyposis colorectal cancer (HNPCC) is between 1:2000 and 1:660, its causal variant should have low frequency in the general population [40]. We obtained 3528 (592) rare non-silent SNVs (indels) after removing the variants whose frequencies were greater than 1%. Then, we focused on HNPCC-associated DNA MMR genes and found that only one rare non-silent variant, rs267607964 (chr2: 47693796: G > T), affects the G504 splicing site of MSH2 (Fig. 2a). Although this site was recorded in the dbSNP database, no previous studies have reported the association of this variant with HNPCC. To obtain more information relating to the function of rs267607964, we further searched databases and the literature and found that its adjacent site rs267607962 (chr2: 47693795: A>G) was reported to be a pathogenic variant of Lynch syndrome in the ClinVar database, which suggested that rs267607964 might also associate with Lynch syndrome.

Next, we examined the genotype of rs267607964 in the blood cells of other family members by Sanger sequencing. As shown in Fig. 2b, the four healthy controls (III1, III5, IV5, and IV7) had G/G genotype, while all cancer patients (III2, III4, and III6) had G/T genotype.

Finally, we examined the effect of MSH2 variation (rs267607964) on protein expression. As shown in Fig. 2c, MSH2 was negative in all tumor samples. Decreased MSH2 protein resulted in defected DNA repair system, which further caused MPC in different tissues. These results support the clinical diagnosis of the patients and revealed the genetic cause of the rare family. Of note, IV3, a 39-year-old woman who developed ovarian cancer at age 30, exhibited G/T variant of MSH2 and decreased MSH2 expression, suggesting that she had a higher risk of suffering additional Lynch syndrome-related cancers in the future.

### Mutation landscape of the patient with Lynch syndrome

**Fig. 3:**
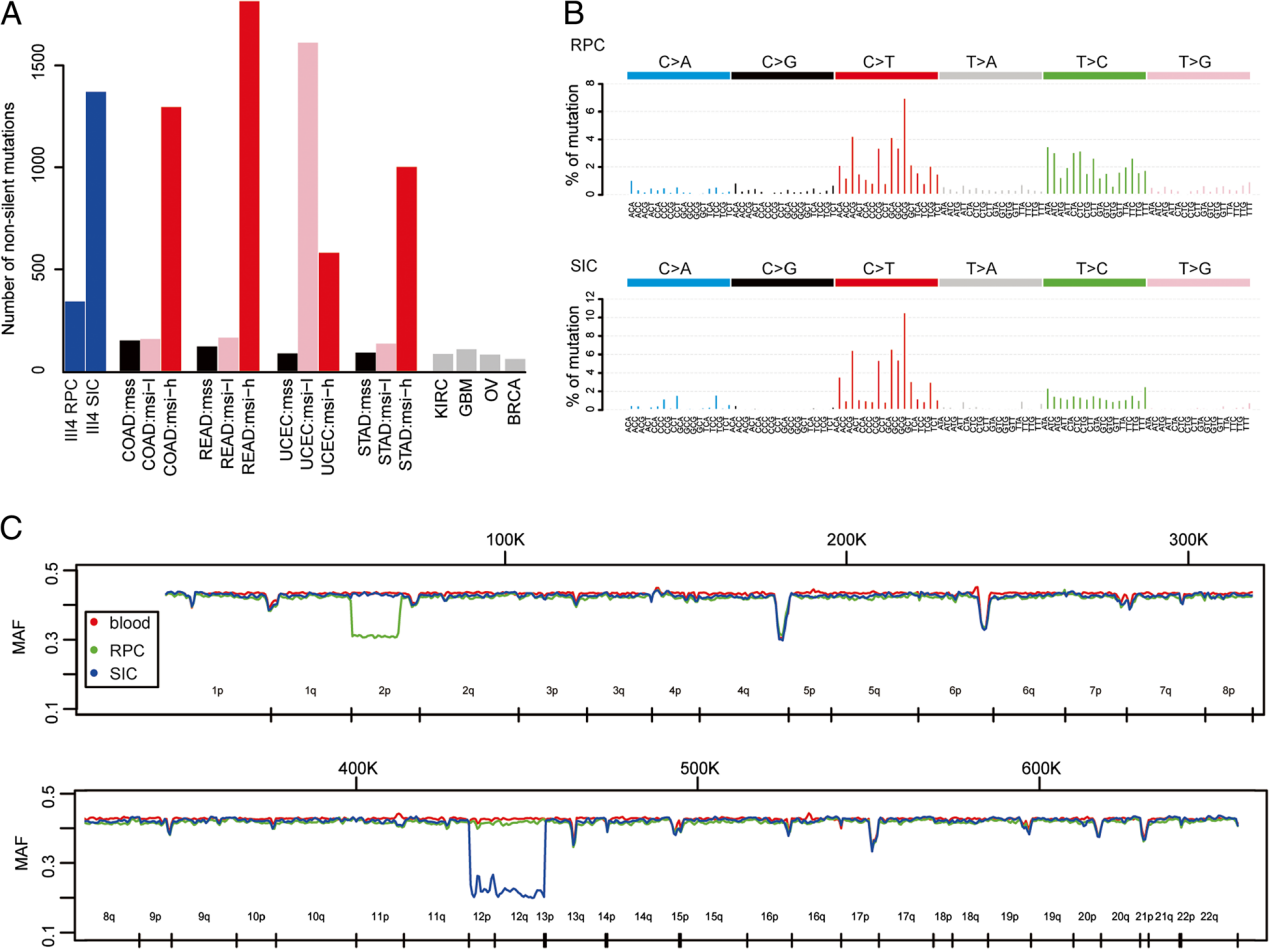
Somatic mutations and copy number alternations in a patient with Lynch syndrome. **a** Number of somatic mutations in the RPC and SIC of the proband. They were compared with another eight cancer types in TCGA database, among which four cancer types had microsatellite instable samples. **b** Mutation pattern of the 96 possible trinucleotide context. **c** Minor allele frequency of germline single nucleotide variants in blood cells, RPC, and SIC

Taking the blood sample as the control, we detected 343 and 1373 non-silent somatic mutations in RPC and SIC of the proband, respectively. The mutation rate of RPC and SIC were significantly higher than the MSS cancers in TCGA (Fig. 3a). A hyper-mutated genome is the typical feature of microsatellite instable cancers.

Next, we investigated the mutation patterns, which may reflect the mutational process during Lynch syndrome development [41, 42]. There were six classes of base substitutions (C>A or G>T, C>G or G>C, C>T or G>A, T>A or A>T, T>C or A>G, and T>G or A>C), which composed 96 possible trinucleotide contexts when considering the adjacent bases. We classified mutations based on the trinucleotide context and counted the number of mutations in each class. We found that mutations in the proband were characterized predominantly by C>T transitions at the NCG context (Fig. 3b). This pattern was compared to the 30 mutational signatures that were found by analyzing 10,952 exons and 1048 whole genomes across 40 distinct types of human cancers (http://cancer.sanger.ac.uk/cosmic/signatures). The mutation pattern of the Lynch syndrome proband was mostly similar to signature 6, which was associated with defective DNA MMR, and it was also found in microsatellite unstable cancers.

Furthermore, we analyzed the somatic copy number alterations (SCNAs) by comparing the minor allele frequency (MAF) curve between tumor and normal samples (Fig. 3c) and found that chr2p was lost in RPC and chr12 was lost in SIC. Loss of heterozygosity (LOH) analysis showed that 2125 LOH sites exist in RPC chr2p and 16,827 LOH sites exist in SIC chr12. The copy numbers of other chromosomes were neutral, which was consistent with previous reports, which showed that microsatellite instability (MSI) colorectal cancers generally have near-diploid karyotypes [43].

### Comparative genome analysis of two cancers in the patient with Lynch syndrome

**Fig. 4:**
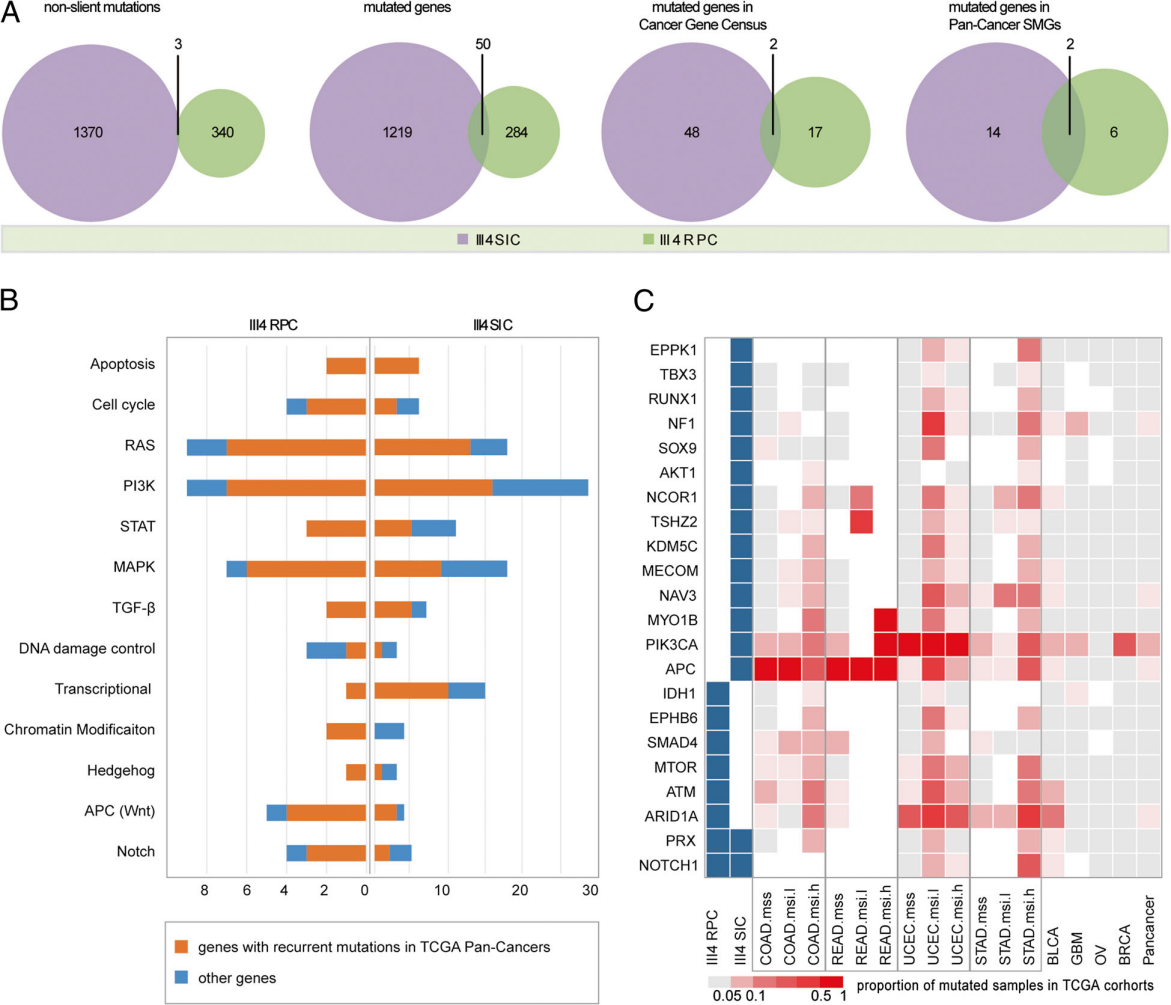
Comparison of altered genes and pathways in different cancer tissues of a patient with Lynch syndrome. **a** Venn diagram showing the overlapping sites or genes in RPC and SIC. **b** Number of altered genes in 13 cancer-related pathways. Orange represents genes with recurrent mutations in TCGA Pan-Cancer Project and blue indicates other genes. **c** Summary of point mutations in 22 genes across 2 cancers from patient III4 and 8 cancer types of TCGA. These 22 genes are mutated in RPC or SIC, and they were reported as significantly mutated genes by TCGA Pan-Cancer analysis

Then, we compared the similarity of mutated sites or genes between RPC and SIC in the proband (Fig. 4a). We observed extremely few overlapping between the two cancers at the whole genome level and cancer-related gene lists. Such overlap was significantly lower than the similarity (30~80%) between paired primary metastasis cancers [44, 45], which might indicate that cancers from different tissue origins have independent mutation landscape after initiation by MSH2 inactivation.

After that, we analyzed the altered genes across 13 major cancer pathways. Figure 4b shows the number of altered genes in RPC and SIC. Genes are red or blue colored according to whether their mutations were recurrent in the TCGA Pan-Cancer dataset. Since cancers in the Lynch syndrome proband were hyper-mutated, each pathway had altered genes. The number of altered genes appears to be randomly distributed and they were not enriched in a specific pathway. NOTCH1, THBS1, and RIN1 were mutated in both RPC and SIC. Furthermore, other recurrent Pan-Cancer genes were not commonly altered in both cancers (Additional file 2: Figure S1).

Finally, we focused on 127 significantly mutated genes (SMGs) that were identified by TCGA Pan-Cancer analysis [46]. Twenty-two SMGs were mutated in RPC and SIC: two SMGs were widely altered in both cancers, six were only mutated in RPC, and fourteen were only mutated in SIC. We compared the mutation frequencies of these 22 genes across 8 TCGA cancer types (Fig. 4c). Different cancer types shared some SMGs and each had type-specific SMGs. MSI cancers had a higher proportion of mutation than MSS cancers did. Since the TCGA project did not include RPC and SIC, and that RPC is close to bladder urothelial carcinoma (BLCA), while SIC is close to colon adenocarcinoma (COAD) based on their location, then BLCA and COAD sequencing data were used for the subsequent analysis. We observed that cancers from the patient with Lynch syndrome harbored mutations in the driver genes of similar TCGA cancers (Additional file 1: Table S4). RPC had missense mutations in two BLCA driver genes [47]: ARID1A (MAF = 0.26), ATM (MAF = 0.30), and MTOR (MAF = 0.18). SIC had missense mutations in two driver genes of COAD [46]: APC (MAF = 0.32) and PIK3CA (MAF = 0.41). Additionally, SIC also harbored a somatic mutation in another MMR gene, MSH3 (MAF = 0.42). The mutation frequencies of driver genes were higher than the majority of mutations. Higher mutant allele frequency suggests that the mutation occurred earlier during cancer evolution. Therefore, we inferred that mutations in these driver genes might be early and necessary events in the carcinogenesis of Lynch syndrome.

### Model of mutation progress

**Fig. 5:**
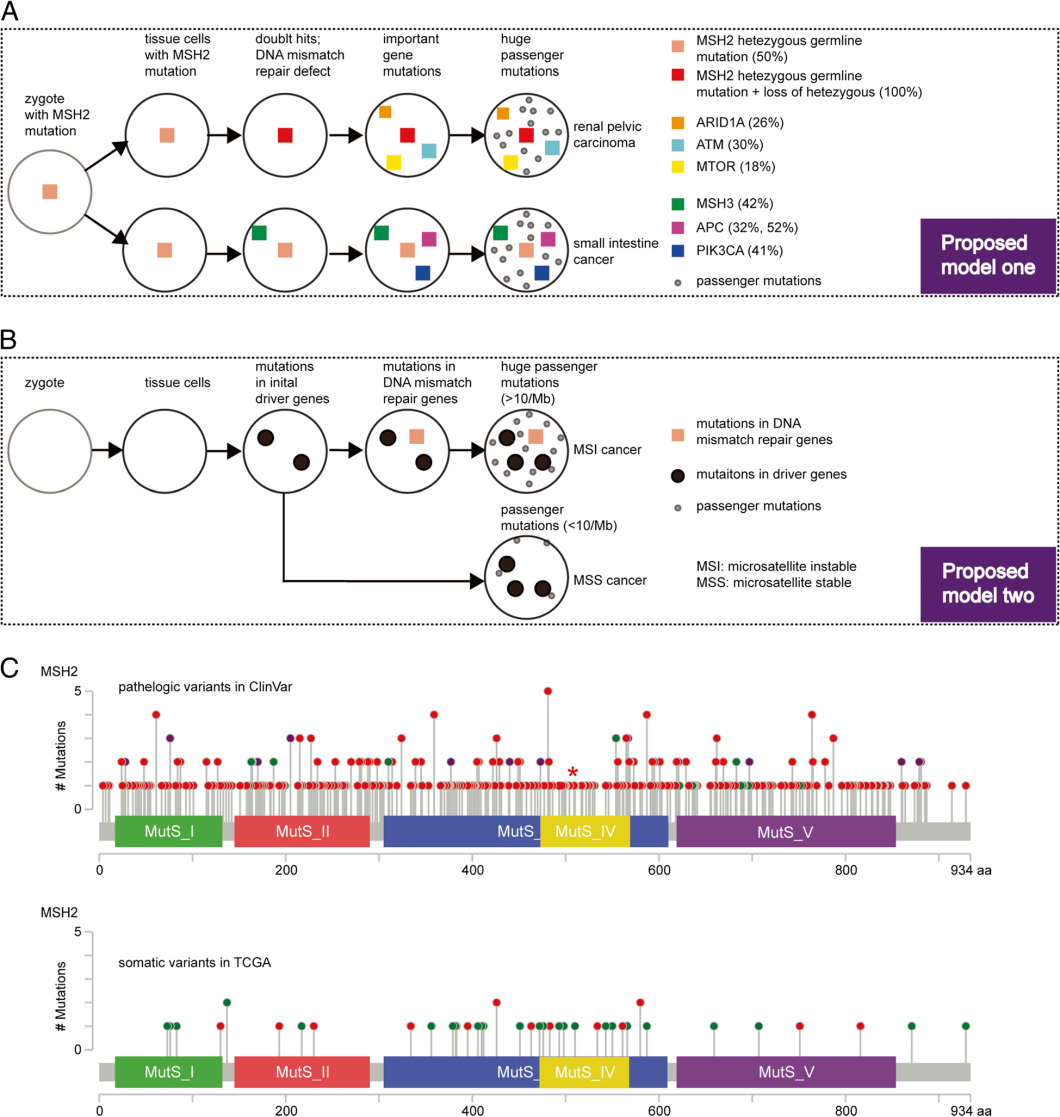
MH2 mutations and its effects in inherited and non-inherited cancers. **a** Proposed model for the mutagenic progress of MPC in Lynch syndrome. **b** Proposed model for the mutagenic progress of non-inherited cancers. **c** MSH2 diagram of the germline variants in inherited cancer syndromes or somatic mutations in TCGA Pan-Cancer. Germline variants were collected from the previous reported pathologic variants in ClinVar database. The mutation found in the present study is highlighted by the asterisk. Red color illustrates truncating mutations (nonsense, nonstop, splicing, frameshift, and indels)

We comprehensively investigated the genomic landscape of the proband from a Chinese family with Lynch syndrome and found a new pathologic germline mutation on MSH2 and revealed important somatic mutations that may drive carcinogenesis. Based on these findings and our previous understanding on Lynch syndrome, we proposed a model of mutation progress in MPC for Lynch syndrome (Fig. 5a). First, an individual inherits a pathologic germline mutation in a MMR gene from his/her parent, with all germline cells carrying this variant. Second, germline mutation results in loss of function of the encoded protein and MMR system is damaged. Sometimes, somatic mutation or methylation may serve as a “second hit” at the wild-type allele or other MMR genes [48, 49], which is consistent with Knudson’s double-hit model of carcinogenesis [50]. Double-hit mutations might cause a more severe cancer in phenotype. Third, the cell accumulates huge somatic mutations, with mutations in

driver genes (oncogene or tumor suppressor gene) playing important roles in carcinogenesis. This model could help to explain the observed mutation landscape of proband III4. All cells of III4 had a heterozygous MSH2 splicing mutation, and MSH2 protein was almost not expressed. In the left renal pelvis, the heterozygous MSH2 mutation became homozygous due to loss of heterozygosity. Somatic mutations of potential driver genes such as ARID1A, ATM, and MTOR promoted cancer growth, and many mutations were generated due to MMR deficiency. Therefore, normal tissue became cancerous via the coordination of germline and somatic mutations. The mutation progress of SIC was similar to RPC with the key somatic mutations occurring in APC, PIK3CA, and MSH3.

Apart from inherited cancer syndromes, MSH2 mutations and loss of function were also observed in some sporadic cancers. Similar to Lynch syndrome, these cancers are hyper-mutated and microsatellite instable. However, the MSH2 mutation in sporadic cancers occurred only in a somatic tissue and more likely occurred after the initial driver mutations. Taking sporadic colon cancer as an example, APC is the most common initial gene mutated in inherited and sporadic colon cancer [51]; patients had MLH1 and MSH2 mutations later and their cancers were microsatellite instable, while other patients did not have mutations in MMR genes and their cancers were MSS [51]. The mutational progress of sporadic cancers is summarized in Fig. 5b.

Next, we compared MSH2 mutation-positive inherited tumors with sporadic tumors. We applied the ClinVar database to obtain MSH2 germline mutations, which were annotated to be pathologic in Lynch syndrome or hereditary cancer predisposing syndrome. The MSH2 somatic mutations were collected from tumors from the TCGA Pan-Cancer Project. In total, we obtained 378 non-silent pathologic germline variants and 46 non-silent somatic mutations (Fig. 5c). Mutations were classified as truncating or missense, with 89.6% pathologic germline variants truncating while only 32.6% somatic mutations were truncating. The pathologic germline variants significantly enriched truncating compared to the missense mutations (*P* = 2.2e−16). Truncating mutations might be more deleterious than missense mutations and produce a more aggressive phenotype [52, 53]. MSH2 germline variants were the genetic cause of inherited cancer syndrome, while somatic mutations might be merely passenger genes relative to the other driver genes in most cases. Therefore, truncating mutations of MSH2 were more likely to cause inherited cancer syndrome. This finding was also appropriate to other MMR genes (Additional file 3: Figure S2).

## Discussion

To date, multiple researches had focused on MPC with the majority of them being descriptive. Since Billroth proposed the diagnostic criteria of MPC [2], a large amount of studies had examined in detail the incidence, origin, and classification of the disease [13, 14, 54]. However, few studies have shown a convincing illustration on how MPC occurs. In the present study, we reported on a patient with Lynch syndrome who could also be diagnosed as MPC. After that, we preformed genome-wide sequencing on the cancer tissues of the patient, and we revealed a novel pathologic mutation on MSH2 associating with Lynch syndrome. Moreover, integrative analysis demonstrated that truncating mutations of MMR genes were significantly enriched in the patient. In addition, systematical comparison of the mutation landscape revealed that the primary cancers of the patients were evolutionarily independent. Based on the data, we proposed a model to illustrate how Lynch syndrome developed into MPC. To the best of our knowledge, this is the first study to investigate Lynch syndrome from the genomics level. Our data adds insights into the pathogenesis of MPC from the genomic level.

Obviously, our data suggested that MSH2 gene was critical during Lynch syndrome progression to MPC. This promoted us to investigate whether MSH2 mutation alone was strong enough to induce the occurrence of Lynch syndrome-related cancers. However, systematical literature review indicated that genetic disorder and dietary and/or environment factors had synergistic effect in promoting cancer initiation in MSH2-defective individuals. For instance, Belcheva A and colleagues indicated that interaction between microbiota and dietary factors tends to reduce the occurrence of colorectal cancer and other cancers in APC (Min/+)MSH2(−/−) mice [55]. Moreover, there was also report that germline ablation of SMUG1 DNA glycosylase causes loss of 5-hydroxymethyluracil- and UNG-backup uracil-excision activities and increases cancer predisposition of Ung−/−Msh2−/− mice [56]. This suggested that other genetic instabilities were also effective in MSH2-defective resultant cancers. These data in combination reminded that appropriate dietary and lifestyle intervention might also be effective in preventing Lynch syndrome progression.

Over the past decades, “omics” studies had achieved many discoveries in various human malignancies. For instance, scientists had obtained the genomic mutation landscape of the major human cancers by genome-wide sequencing. Bioinformatics analysis suggested that a typical tumor has two to eight “drive gene” mutations, which manifest selective growth advantage, while others are passenger mutations [37]. The mutation rate varies from one cancer to another, with an average of 1/Mb, which increases to 10/Mb in MSI tumors [35]. Additionally, single-cell sequencing and multi-region sequencing were used to infer tumor progression, which largely extends our knowledge of carcinogenesis. Unfortunately, these technologies had rarely been used to explore the initiation and progression of MPC. Genetic testing of MMR genes has been widely applied to aid the diagnosis of Lynch syndrome. As a major member of MMR genes, MSH2 exhibits a novel mutation in our analysis. MSH2 functions to repair DNA replication errors, whose dysfunction results in accumulated mutation of the cells and, finally, cancer. We collected known pathologic mutations of hereditary tumors from public databases and analyzed the association between MSH2 mutation spectrum and Lynch syndrome. We showed that mutations within the entire length of the coding sequence of MSH2 were positively correlated with Lynch syndrome, which suggested that the mutation of MSH in our study is a novel pathogenic factor. The mutation pattern of MSH2 was further studied by comparing germline mutations in inherited tumors with somatic mutations in sporadic tumors. We found that truncating mutations were more likely to be causal in hereditary Lynch syndrome than missense mutations. This will assist in the annotation of pathogenicity of MMR genetic variants.

Patients with Lynch syndrome tend to develop cancers in multiple tissues, such as colorectal, pancreas, stomach, and so on. However, it is still unclear whether they are related and share the similar mutational landscape. Moreover, the mutation landscapes of the cancers of the proband indicated that they developed independently. This supported the fact that multiple cancers in Lynch syndrome are primary but not metastasis.

Of note, we proposed a double-hits theory during Lynch syndrome progression to MPC in our study. As put, the first hit was the genetic mutation of MSH2, and the second hit was caused by somatic mutations. The second hit, including the loss of heterozygosity at the MSH2 mutation site in the renal pelvic and a new MSH3 somatic mutation in the small intestine might be distinct among tumor tissues. A previous study reported the loss of the wild type MLH1 gene in hereditary nonpolyposis colorectal cancer [48]. These results suggested that double hits of DNA MMR genes might be a common event in the development of other malignancies in Lynch syndrome patients.

Even more interesting is that some SMGs of sporadic tumors also had high-frequency mutations in Lynch syndrome-related cancers. Higher alternative allele frequency indicates that SMG mutations were not a random event, and they might occur earlier than other passenger mutations. This finding highlights a potential role of SMGs in the carcinogenesis of Lynch syndrome. Based on these data, we proposed a mutation progress model of MPC in Lynch syndrome, which include germline mutations of MMR genes, double hits of MMR system, mutations in tissue-specific driver genes, and rapid accumulation of additional passenger mutations. This model may advance the elucidation of carcinogenic theory. Although this model was established based only on a single patient, it was consistent with our prior knowledge of Lynch syndrome.

## Conclusion

In conclusion, we proposed the notion that Lynch syndrome patients with more than two cancers belong to a special type of MPC. We studied a Chinese patient with Lynch syndrome from whole genome level and found that MPC evolves from different somatic mutation progresses and that both genetic and somatic alterations are the driving forces of carcinogenesis. Based on these data, we proposed a model to illustrate how Lynch syndrome progressed into MPC. Our findings extend the knowledge of Lynch syndrome and help to advance the elucidation of carcinogenic theory of MPC.

## Additional files

Additional file 1: Table S1. Clinical description of cancer patients. **Table S2.** Statistics of whole-genome sequencing results. **Table S3.** SNVs and indels that called by GATK and passed the quality control. (DOCX 90 kb)

Additional file 2: Figure S1. Summary of altered genes in 13 cancer related pathways. (TIFF 3983 kb)

Additional file 3: Figure S2. Clinical description of cancer patients. Table S2 Statistics of whole-genome sequencing results. Table S3 SNVs and indels called by GATK and passed the quality control. (TIFF 5406 kb)

## Abbreviations

FAP: Familial adenomatous polyposis
MMR: Mismatch repair
MPC: Multiple primary cancer
RPC: Renal pelvic carcinoma
SIC: Small intestine cancer
TCGA: The Cancer Genome Atlas
