# Supplementary material for: Association of a novel point mutation in MSH2 gene with familial multiple primary cancers
Source: J Hematol Oncol. 2017 Oct 3;10:158. doi: 10.1186/s13045-017-0523-y (PMC5627420; doi:10.1186/s13045-017-0523-y)
Supplement: Supplementary file 1 — Clinical description of cancer patients. Table S2. Statistics of whole-genome sequencing results. Table S3. SNVs and indels that called by GATK and passed the quality control. (DOCX 90 kb) [file 13045_2017_523_MOESM1_ESM.docx]

**Table S1. Clinical description of cancer patients**

| **Individual** | **Gender** | **Age** | **Primary cancers** | | | |
| --- | --- | --- | --- | --- | --- | --- |
|  |  |  | **Order** | **Cancer type** | **Age at diagnosis** | **Stage** |
| III4 (proband) | Female | 61 | 1^st^ | Right Ureteral Transitional Cell Papilloma | 37 | 0 |
|  |  |  | 2^nd^ | Left Breast Infiltrating Ductal Carcinoma | 49 | IB |
|  |  |  | 3^rd^ | Endometriosis type adenocarcinoma | 51 | I |
|  |  |  | 4^th^ | Left renal pelvic infiltrating urothelial carcinoma (RPC) | 58 | I |
|  |  |  | 5^th^ | Small Intestine Ulcerative Infiltrative Adenocarcinoma (SIC) | 59 | I |
| III2 | Female | 67 | 1^st^ | Ascending Colon papillary Adenocarcinoma | 53 | I |
|  |  |  | 2^nd^ | Hypophysoma | 65 | / |
| III6 | Male | 57 | 1^st^ | Transverse Colon tubular Adenocarcinoma | 43 | I |
|  |  |  | 2^nd^ | Poorly differentiated cardia carcinoma | 55 | IA |
| IV3 | Female | 39 | 1^st^ | Ovarian Cancer | 30 | IA |
| II1 | Male | / | / | Nasopharyngeal Carcinoma | / | / |
| II3 | Female | / | / | Malignant Glioma | / | / |
| II4 | Female | / | / | Esophageal Cancer | / | / |
| I2 | Female | / | / | Cervical Cancer | / | / |
| I3 | Male | / | / | Esophageal Cancer | / | / |

**Table S2. Statistics of whole-genome sequencing results**

| **Individual** | **Raw data (billion bases)** | **High-quality data (%)** | **Aligned reads (%)** | **Mean depth (X)** |
| --- | --- | --- | --- | --- |
| III4 blood | 120.2 | 99.85 | 99.7 | 41.2 |
| III4 RPC | 117.7 | 99.33 | 99.6 | 34.9 |
| III4 SIC | 115.3 | 99.34 | 99.6 | 36.3 |

**Table S3. SNVs and indels that called by GATK and passed the quality control**

| **Individual** | **SNVs** | **Heterozygous/Homozygous ratio of SNVs** | **Transition/Transversion ratio of SNVs** | **Indels** |
| --- | --- | --- | --- | --- |
|  |  |  |  |  |
| III4 blood | 3,706,485 | 1.37 | 2.05 | 692,967 |
| III4 RPC | 3,303,897 | 1.25 | 2.04 | 609,014 |
| III4 SIC | 3,462,703 | 1.32 | 2.08 | 921,389 |
