# Supplementary figures and images for: Association of a novel point mutation in MSH2 gene with familial multiple primary cancers
Source: J Hematol Oncol. 2017 Oct 3;10:158. doi: 10.1186/s13045-017-0523-y (PMC5627420; doi:10.1186/s13045-017-0523-y)

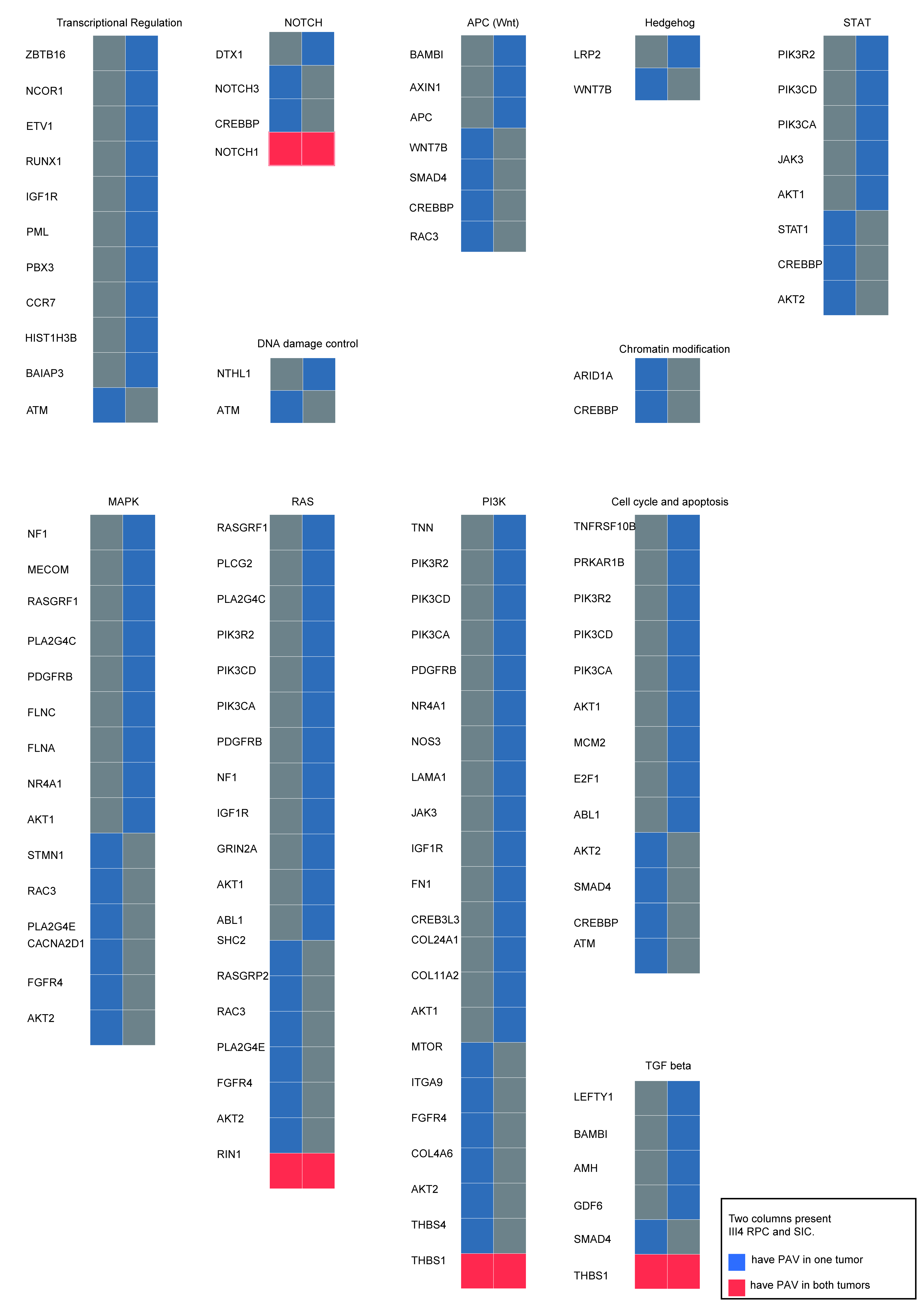

Supplement: Supplementary file 2 — Summary of altered genes in 13 cancer related pathways. (TIFF 3983 kb) [file 13045_2017_523_MOESM2_ESM.tif]

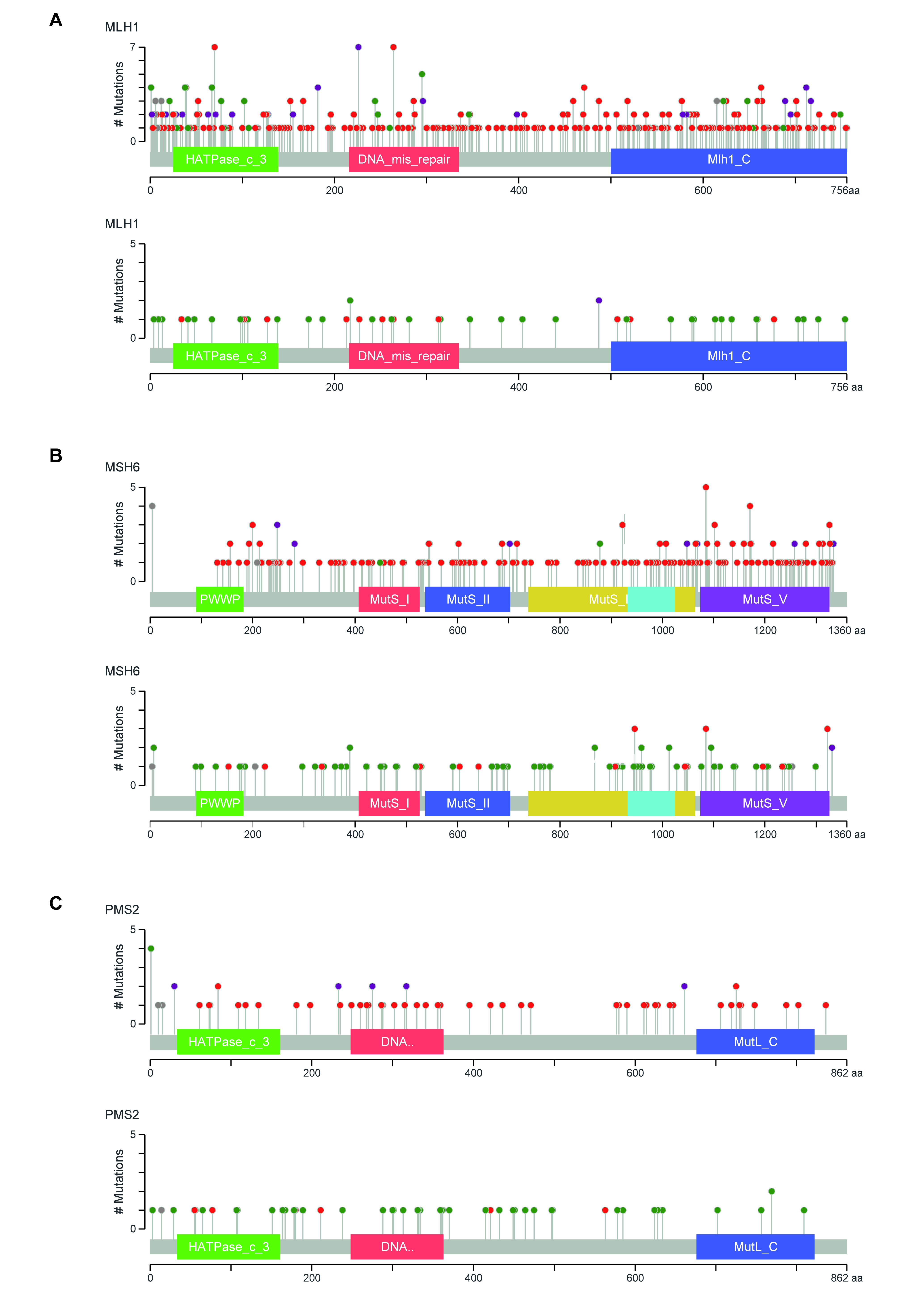

Supplement: Supplementary file 3 — Clinical description of cancer patients. Table S2 Statistics of whole-genome sequencing results. Table S3 SNVs and indels called by GATK and passed the quality control. (TIFF 5406 kb) [file 13045_2017_523_MOESM3_ESM.tif]
